# Radiomics in lung cancer for oncologists

**Published:** 2020-09-02

**Authors:** Carolina de la Pinta, Nuria Barrios-Campo, David Sevillano

**Affiliations:** ^1^Department of Radiation Oncology, Ramón y Cajal Hospital, Madrid, Spain; ^2^Department of Biomedical Engineering, Madrid Polytechnic University, Madrid, Spain; ^3^Department of Medical Physics, Ramón y Cajal Hospital, Madrid, Spain

**Keywords:** radiomics, radiogenomics, lung cancer, diagnosis and response

## Abstract

Radiomics has revolutionized the world of medical imaging. The aim of this review is to guide oncologists in radiomics and its applications in diagnosis, prediction of response and damage, prediction of survival, and prognosis in lung cancer. In this review, we analyzed published literature on PubMed and MEDLINE with papers published in the last 10 years. We included papers in English language with information about radiomics features and diagnostic, predictive, and prognosis of radiomics in lung cancer. All citations were evaluated for relevant content and validation.

**Relevance for patients:**

The evolution of technology allows the development of computer algorithms that facilitate the diagnosis and evaluation of response after different oncological treatments and their non-invasive follow-up.

## 1. Introduction

Lung cancer is the first cause of cancer in the world, constituting the first cause of cancer mortality [1]. There are two types of lung cancer, non-small cell lung cancer (NSCLC) and small cell lung cancer (SLC). NSCLC includes adenocarcinomas and epidermoid carcinomas.

The diagnosis of lung cancer must be histological, but sometimes it is not possible because the lesion is not accessible or the diagnostic procedure represents a high risk for the patient. Sometimes, the oncological treatment can be planned despite the lack of histological diagnosis. Surgery and radiotherapy are locoregional treatments that contribute, alone or in combination with other treatments, to the cure of early stages lung cancer. In more advanced stages, when metastases are present, chemotherapy and other systemic treatments are usually the treatment of choice. Stereotactic body radiotherapy, or SBRT, is a high precision irradiation technique, which allows very high doses to be delivered to the tumor in a limited number of fractions, with a highly cytotoxic biological ablative effect, and minimal doses to the organ at risk [2]. The use of SBRT to treat early-stage lung cancer has become increasingly due to proven efficacy, demonstrating local control at 3 years of up to 98% with limited relevant toxicity [3].

Radiomics was introduced by Lambin *et al*. [4] in 2012, but Kumar *et al*. [5] extended the definition. Radiomics allows the conversion of digital medical images into data to extract the underlying physiopathology. Radiomics was designed to decode the information and characteristics of lesion images to improve their interpretation. It is a product of digital imaging combined with various computer techniques. The use of this tool could provide information to improve medical decision-making and screening programs [6]. In addition, it could be an appropriate non-invasive tool to determine malignancy, tumor subtype, stage tumors, treatment, or predict response, relapses, and prognosis. For its development, radiologists, medical experts, mathematicians, and computer scientists are necessary. Radiomics can be extracted from CT, MRI, and PET imaging [7].

## 2. Radiomic Features: Data Extraction and Analysis

Radiomics analysis allows the evaluation of medical images. The workflow includes image acquisition and reconstruction; definition of region of interest (ROI) and segmentation; extraction and quantification of characteristics; and finally, construction of predictive and prognostic models with the information identified [7,8].

### 2.1. Image acquisition and reconstruction

Image acquisition is important in the final results of the analysis. Standardization of image data can help to establish predictive models. Characteristics include kV, mAs, slice size, breath control method, configuration, and contrast. Variations in image acquisition and reconstruction parameters can affect the image value to compare and analyze radiomics studies. It is very important to maintain a homogeneous criterion [9]. Radiomics based on non-contrast CT images has shown in some studies a higher efficiency compared to contrast CT images. He *et al*. [10] suggested that biological heterogeneity within the tumor, represented by radiological features, may be confused with intravenous contrast agents, leading to inferior discrimination between benign and malignant tumors.

### 2.2. Segmentation of the area of interest

Once the image is obtained, the ROI, the area of the tumor, is delimited. This process is denominated segmentation. Segmentation can be manual, automatic, or semi-automatic. The gold standard includes manual segmentation by experts but is operator dependent. The automatic segmentation uses pre-selected parameters and is ideal for its accuracy, reproducibility, and consistency. However, manual intervention is necessary to validate the automatic segmentation. There is no universal method, the same algorithm can give variable results. The semiautomatic segmentation is able to combine two previous procedures being the most recommended. Segmentation is crucial, an error in this phase will modify the whole analysis [11-13].

### 2.3. Extraction and quantification of features

It allows to extract and evaluate large volumes of data obtained from the images. Radiomics includes four types of analysis: Morphological, statistical, regional, and model-based. The most basic radiomics analysis is morphological analysis. Statistical analysis includes first-order and highly-order features, histogram, and texture, respectively. Regional analysis includes intratumor heterogeneity and characteristics around the tumor. Model-based is analyzed with a mathematical approach.

Morphological analysis includes information about shape [14]. Shape allows to evaluate physical characteristics of the tumor and differentiates between malignant and benign nodules [15]. Shape parameters include diameter, volume, area under the curve (AUC), and wave [16]. To study the shape, the segmented lesions are constructed in 3D images. The most commonly used parameters are the maximum and minimum diameter and volume. The volume is defined by counting the number of voxels in the tumor and multiplying by the volume of the voxel. Volume is a key parameter, a short volume doubling time reflects high histological aggressiveness and suggests poor prognosis [17,18]. Indeed, volume is a tool for evaluating response to treatment [19,20].

Intensity is analyzed in histograms which are graphic representations of the intensity distribution in an image. Intensity analysis includes range, mean, median, standard deviation (SD), minimum, maximum, kurtosis, energy, entropy, uniformity, variance, and skewness and can be used to predict the nature of the lesion and prognosis. Intensity characteristics are dependent on the reconstruction and image acquisition parameters (cut size and voxel size) [19]. Texture describes the relationship between neighboring pixels and their distribution through the nodes. The texture determines the tumor’s heterogeneity, which is very important in the aggressiveness evaluation, allowing the differentiation between benign and malignant lesions. For texture extraction, the most used method includes second-order statistics and co-occurrence matrix characteristics constructed using number, distance, and angle of gray levels in the image. Texture parameter includes correlation, clustering, contrast, energy, and entropy. Entropy describes the randomness of the surrounding intensities within a grayscale image. Wavelet allows to decompose the image data into different frequency components and uses these data to extract characteristics related to the texture and intensity of the image. These are filters that transform an array of complex lines or radio waves. The most common is the Coiflet wave transformation [21]. They are used in the diagnosis and evaluation of response to treatment. It is necessary to standardize it.

The relationship between the tumor and the surrounding healthy surface is another element of the tumor microenvironment. The discrete compaction is related to its circularity and this to the invasion around the tumor [22]. The neighboring gray tone matrix is a parameter to differentiate gray tones, including busyness, complexity, and texture length. It is necessary to evaluate these data with statistical co-variance [7]. There are several calculation algorithms; one example is in neighborhood gray tone various matrices, which analyzes the intensity values around and within the pixels, and has been shown to be a predictor of survival in patients with NSCLC or the gray level co-occurrence matrix that uses the distance and angle of a combination of grays that occur in an image [19]. Aerts *et al*. [20] found a great correlation between gene expression and textures. Cook *et al*. [22] analyzed texture to predict survival after chemoradiotherapy (CRT) in lung cancer. Ganeshan *et al*. [23] found that tumor heterogeneity can be evaluated by non-contrast CT texture analysis and has the potential to provide an independent predictor of survival and prognosis for patients with NSCLC. In general, several studies have related texture with stage, metastasis, response, survival, and metagenesis in lung cancer [22,24]. This is a promising prognostic indicator.

### 2.4. Construction of predictive models and prognosis in a non-invasive method

Once the characteristics have been extracted, the next step is to establish relationships between radiomics parameters and clinical variables. This can be done from direct statistical analysis based on hypotheses on machine learning methods. Statistical analysis is the most commonly used in lung cancer. One of the predictive models developed combines size, concavity, contour, and speculation [25]. Bayanati *et al*. demonstrated that size can be a good predictor in itself and correlates with overall survival (OS) [26]. This model can be used to define not only the nature of the primary tumor but also the nodes [26,27].

## 3. Radiomics Applications in Lung Cancer

### 3.1. Diagnostic prediction: malignant versus benign histology

Images in oncology can be predictive and diagnostic. In lung cancer screening, correct diagnosis is essential. Some studies have reported that radiomics is useful for differentiating NSCLC from other benign tumors or pre-malignant lesions by extracting characteristics of solid nodules [28]. The differentiation between benign nodules from malignant, solid regions from sub-solids, or adenocarcinoma *in situ* from invasive is essential for the correct treatment of lung cancer. Previously, discrimination between invasive and non-invasive proportions of ground-glass opacity (GGO) nodules was limited to visual perception and subjective CT analysis. In the radiomic era, some studies have shown that entropy and a high attenuation value are factors of invasiveness in adenocarcinoma [29]. In addition, the 97.5 percentile and slope of CT attenuation have been defined as predictors of changes in attenuation and growth of the solid zone of the GGO node, providing additional information on invasiveness [30].

In lung adenocarcinoma, the increase in cellularity is important because it reflects the presence of invasiveness [29]. Coroller *et al*. have differentiated pre-invasive tumors from invasive adenocarcinomas using texture, high kurtosis, and small nodules [35]. Gradient characteristics extracted in various resolutions and orientations provide nodal information for detection and diagnosis [31]. Variability in nodule size and orientation relating to the lung adversely affects the gradient [32]. The study of tumor heterogeneity allows the differentiation of tumor aggressiveness [14].

Radiological analysis of the primary helps predict lymph node involvement. Yang *et al*. [33] built a model that correlated different characteristics with nodal involvement, with an AUC of 0.871 in the training group and an AUC of 0.856 in the validation cohort. Zhong *et al*. [34] studied 300 radiological characteristics in 492 patients with lung adenocarcinoma. The accuracy of the radiomic signature was 91.1%, suggesting that the radiomic signature of the primary tumor can be used for quantitative and non-invasive prediction of lymph node metastases in patients with lung cancer [21].

However, few studies have studied radiomic features of lymph nodes. Bayanati *et al*. [26] found that texture and shape identified malignant lymph nodes with a sensitivity of 81% and a specificity of 80%. Andersen *et al*. [27] demonstrated that texture analysis showed a significant difference between malignant and benign lymph nodes with an AUC of 83.4% and excellent reproducibility. Interestingly, Coroller *et al*. [35] performed analyses on both the primary tumor and the lymph nodes and demonstrated that the lymph node phenotype could present essential information in addition to that provided by the primary tumor alone.

We should note that the studies focus on only one primary ROI per subject. There are many cases in which a patient has multiple nodes, which may be primary or metastatic. Focusing on a single ROI facilitates statistical analysis, but does not include the interactions among the different nodules; this is currently under development [36].

### 3.2. Prediction of response: Non-invasive monitoring tool

After treatment, patients are monitored to assess the response to treatment and possible progressions and complications. This follow-up is usually done with imaging tests such as CT or PET. Response evaluation is through Response Evaluation Criteria in Solid Tumors (RECIST) criteria. RECIST allows for standardized evaluation of size differences in serial CT scans after treatment to define the response. It has been adapted to the new therapies with immune-related response criteria and modified RECIST; however, they are still not sufficient for the evaluation of the accurate response in certain scenarios. Sometimes, large inflammatory changes are described by treatments such as SBRT or immunotherapy. Radiomics has shown to be a promising tool in predicting response to treatment. There are several radiomic features related to this response such as tumor shape and textural patterns. It has been studied in the evaluation of early response and treatment effectiveness.

Some parameters studied in PET include standardized uptake value (SUV), metabolic tumor volume (MTV), and quantitative texture characteristics such as entropy, correlation, contrast and uniformity, and delta radiomics (volume, texture, and intensity-volume histogram). Of these, texture by coarseness, contrast, and busyness present a strong correlation with response to CRT with RECIST, while SUV does not. Dong *et al*. [37] demonstrate a correlation among the coefficient of variation, MTV, and contrast with the prediction of response to CRT.

In CT findings such as pre-treatment spherical shape, texture data, lymph node homogeneity, changes in primary tumor volume, and histogram characteristics are potential predictors of patients with NSCLC after CRT, Coroller *et al*. [35] demonstrated that radiomic wavelet features predicted pathological complete response in patients treated with CRT. Jain *et al*. [38] demonstrated that textured features predicted pathological complete response in patients treated with trimodal therapy. Fave *et al*. [39] demonstrated that changes in intensity and texture of serial CT scans of stage III patients before, during, and after radiation therapy were predictive of tumor response. Variability in intensity and size is predictive of response after tyrosine kinase inhibitors [40].

In predicting response after SBRT, the use of shape and tumor heterogeneity features has been shown to be predictive [41]. Mattonen *et al*. [42] found an increased gray-level co-occurrence and gray level features in patients who recurred after early-stage NSCLC radiotherapy.

### 3.3. Predicting radio-induced lung injury (RILI)

Distinguishing radio-induced damage from local recurrence is imperative. Mattonen *et al*. [42] demonstrated that, compared to RILI, recurrence showed higher Hounsfield units (HU) and higher SD in the texture of the GGO nodule. When comparing subjective study characteristics and quantitative changes, results showed that the key to distinguish RILI and early recurrence is time, 9 and 15 months after SBRT, respectively. Mattonen *et al*. [42] described that GGO texture analysis can predict recurrence within 5 months after SBRT. Moran *et al*. [43] extracted first-order and gray-level texture characteristics to distinguish the severity of RILI in three categories. They demonstrated that texture characteristics provide better performance than first-order characteristics. Cunliffe *et al*. [44] also combined radiomic characteristics with radiation dose and demonstrated that radiology can provide a quantitative and customized measure of radiation dose tolerance for each patient, which can be used to determine the likelihood of radiation-induced pneumonitis.

### 3.4. Predicting risk of recurrence

Takeda *et al*. [45], Essler *et al*. [46], and Zhang *et al*. [47] evaluated local recurrence in NSCLC after SBRT on PET images, with the SUV being a strong predictor with low variability. Pyka *et al*. [48] related the risk of recurrence to texture and entropy in CT in patients treated with radiotherapy. In addition, wavelet and textural features were overexpressed in patients with distant metastases who failed with SBRT. With respect to PET/CT scans, Li *et al*. [49] explored the SUV use of lymph nodes compared to primary tumor characteristics and reported that lymph node characteristics added value in predicting relapse.

### 3.5. Predicting prognosis and survival

Grove *et al*. [50] found measures of heterogeneity as speculation and entropy indicators of prognosis of OS. Ganeshan *et al*. [23] and Win *et al*. [51] found textural features correlated with OS, but Fried *et al*. [24] reported that texture is not correlated with OS, but it is correlated with locoregional control and disease-free survival (DFS); Song *et al*. [52] described wavelet correlated with OS, and Huang *et al*. [53], Raghunath *et al*. [54], and Depeursinge *et al*. [55] found a correlation between radiomic biomarkers in CT and progression-free survival (PFS). Parmar *et al*. [21] found the association of size, intensity, shape, texture, and wavelet with prognosis in lung cancer, stage, and histology and Coroller *et al*. [38] found a signature that correlated with distant metastasis.

The texture is correlated in studies with heterogeneity and aggressiveness.

Radiomics in PET is associated with survival, including OS, PFS, locoregional free survival, and distant metastases free survival [56]; and mean SUV max of 3.45 for DFS [50]. Delta radiomics of FDG PET correlates with OS in patients with NSCLC, as demonstrated by Carvalho *et al*. in their study [57].

CT seems to be better than PET for predicting OS. Some studies have demonstrated that the combination of shape, intensity, texture, and delta radiomics with clinical factors improves the predictive capacity.

Of all the studies in this review, only one showed a relationship between the intensity parameter and survival [15]. In addition, only one study used daily imaging of radiotherapy treatment and found that reduction in HU tumor correlated with cumulative dose and was associated with survival [58].

The most relevant studies in radiomics prediction of survival and local control are summarized in Table 1.

**Table 1:** Radiomics for prediction survival and local control.

Study	n	Stage	Treatment	Image	Radiomic Features
Survival					
Huang^52^	282	I, II	-	CT	DFS: Texture
Parmar^23^	464	NSCLC	Pre-treatment	CT	OS: Size, intensity, shape, texture, wavelet
Coroller^61^	182	NSCLC	CRT	CT	DMFS: First order statistics, texture, wavelet
Song^51^	199	NSCLC	-	CT	OS: Wavelet
Fried^21^	91	III	RT	CT/PET	OS, LC, DMFS: Textura+clinical parameters
Ganeshan^22^		I-IV NSCLC	Pre-treatment	CT	OS: Texture
Depeursinge^54^	101	I, AC, resected	Surgery	CT	DFS: Texture
Grove^49^	108	Early	-	CT	OS: Shape, texture
Balagurunathan^62^	59	NSCLC	-	CT	OS: Shape, texture
Carvalho^63^	220	I-IIIB	CRT	PET	OS: Delta radiomics (volume, texture and intensity-volume histogram)
Li^64^	92	I-IIA	SBRT	CT	OS: Morphology and clinical parameters
Aerts^19^	647	CRT	CT	OS: Shape, intensity, texture
Song^65^	152	I-IV	TKI	CT	OS: Wavelet
Huynh^39^	113	I, II	SBRT	CT	OS: First order statistics, texture, shape Metastases: Wavelet
Local control					
Mattonen^40^	45	Recurrence	SBRT	CT	Texture
Pyka^48^	45	I-IIA	SBRT	PET	Entropy
Lovinfosse^66^	63	I-II	SBRT	PET	Texture
Wu^67^	101	I	SBRT	PET	Radiomics and histology
Cook^20^	53	IB-III	CRT	PET	Coarseness, contrast, busyness
Kang^55^	116	III	CRT	PET	SUVmax, AUC-CSH
Vaidya^68^	27	IV	SBRT	CT/PET	IVH in PET, COV in CT

CT: computed tomography; DFS: disease free survival; NSCLC: non-small cell lung cancer; CRT: chemoradiotherapy, OS: overall survival; RT: radiotherapy; PET: positron emission tomography; LC: local control; DMFS: distant metastases free survival; AC: adenocarcinoma; SBRT: stereotactic body radiotherapy; TKI: tirosin kinasa inhibitors; SUV: standardized uptake value; AUC-CSH: area under curve of the cumulative SUV-volume histogram; IVH: intensity volume histogram ; COV: coefficient of variation

## 4. Radiogenomics

Genomics is central to targeted treatment of lung cancer. Radiogenomics is the relationship between radiomic phenotypes and genomic information. In general, studies have shown promising results using radiomics to identify radiographic tumor phenotypes that favor specific gene expressions. It is, therefore, essential to study the relationship between genomics and radiomics. Recent studies have studied the mutational state of tumors and radiomic signatures. Aerts *et al*. [9] applied this concept to lung cancer by studying how radiomic data in pre-treatment CT could non-invasively estimate mutations in EGFR, using volume, texture, and gradient. While some groups have found correlations between mutations and radiomic features, these results are not always consistent. Yipp *et al*. [59] scanned radiomic signatures on PET and found that they could detect mutations in EGFR, being unable to detect correlation with KRAS. ALK, ROS1, and RET were associated with kurtosis and inverse variance in combination with clinical radiology in another study [60,61]. Radiogenomics in lung cancer is in development. The scientific community needs large studies and the integration of medical imaging, genomics, and clinical data.

## 4.1. Limitations

Our review has limitations, including the analysis of retrospective studies and the heterogeneity of these studies. Indeed, another limitation was the number of articles reviewed and the criteria to choose which articles to analyze.

## 5. Conclusions

Radiomics is a promising non-invasive tool for the diagnosis and clinical management of lung cancer. Radiomics provides a more adequate and reproducible measurement of the tumor than other previously known methods to evaluate response. Furthermore, the combination of radiomics and genomics has a promising future. However, image acquisition protocols and radiomic analysis systems need to be standardized. More studies are needed to consolidate the data available.
